# Modulation of Cystatin C in Human Macrophages Improves Anti-Mycobacterial Immune Responses to *Mycobacterium tuberculosis* Infection and Coinfection With HIV

**DOI:** 10.3389/fimmu.2021.742822

**Published:** 2021-11-18

**Authors:** David Pires, Marta Calado, Tomás Velez, Manoj Mandal, Maria João Catalão, Olivier Neyrolles, Geanncarlo Lugo-Villarino, Christel Vérollet, José Miguel Azevedo-Pereira, Elsa Anes

**Affiliations:** ^1^Host-Pathogen Interactions, Research Institute for Medicines, iMed-ULisboa, Faculty of Pharmacy, Universidade de Lisboa, Lisbon, Portugal; ^2^Institut de Pharmacologie et Biologie Structurale, IPBS, Université de Toulouse, Centre National de la Recherche Scientifique (CNRS), Toulouse, France

**Keywords:** cystatins, cathepsins, tuberculosis, HIV/Mtb coinfection, host-directed therapies

## Abstract

Tuberculosis owes its resurgence as a major global health threat mostly to the emergence of drug resistance and coinfection with HIV. The synergy between HIV and *Mycobacterium tuberculosis* (Mtb) modifies the host immune environment to enhance both viral and bacterial replication and spread. In the lung immune context, both pathogens infect macrophages, establishing favorable intracellular niches. Both manipulate the endocytic pathway in order to avoid destruction. Relevant players of the endocytic pathway to control pathogens include endolysosomal proteases, cathepsins, and their natural inhibitors, cystatins. Here, a mapping of the human macrophage transcriptome for type I and II cystatins during Mtb, HIV, or Mtb-HIV infection displayed different profiles of gene expression, revealing cystatin C as a potential target to control mycobacterial infection as well as HIV coinfection. We found that cystatin C silencing in macrophages significantly improves the intracellular killing of Mtb, which was concomitant with an increased general proteolytic activity of cathepsins. In addition, downmodulation of cystatin C led to an improved expression of the human leukocyte antigen (HLA) class II in macrophages and an increased CD4^+^ T-lymphocyte proliferation along with enhanced IFN-γ secretion. Overall, our results suggest that the targeting of cystatin C in human macrophages represents a promising approach to improve the control of mycobacterial infections including multidrug-resistant (MDR) TB.

## Introduction

Tuberculosis (TB) is a transmittable disease caused by *Mycobacterium tuberculosis* (Mtb), a pathogen that latently infects about a quarter of the world’s population. From the latently infected group, about 600,000 people are estimated to be carriers of multidrug-resistant (MDR) and extensively drug-resistant (XDR) Mtb strains (1). Coinfection with HIV is highly prevalent and constitutes simultaneously a major risk factor for TB activation from latency and an enormous public health threat by contributing to the spread of MDR–XDR strains (1, 2). Therefore, it is urgent to develop new therapeutic strategies to control the infection and overcome drug resistance.

Macrophages (Mø) are important immune cells in the pathobiology of TB. These cells play a dual role as the principal niche for Mtb persistence and as the main effector cell against the bacilli. Despite the fact that CD4^+^ T cells are the main target for HIV, Mø also constitute relevant viral reservoirs in the context of the lung environment (3), particularly during coinfection with Mtb (4). Both pathogens alter the Mø microbicidal functions converting these phagocytes into cellular reservoirs (5), and they modify the lung immune environment to one more favorable to pathogen replication. In fact, alveolar Mø can be simultaneously infected with HIV and Mtb as demonstrated in coinfected patients (6). In addition, the long-term survival of infected lung Mø turns these reservoirs into a serious challenge for pathogen eradication. For instant, Mø were shown to continue producing HIV in the lung despite antiretroviral therapy, a situation that might be exacerbated in the context of TB-associated microenvironments (7, 8).

Phagocytosis of Mtb by Mø in the lungs is an opportunity for destruction of the bacteria by phagosome fusion with lysosome, exposing the pathogen to lysosomal hydrolases. However, Mtb manipulates these events leading to its survival within vesicles of the endocytic/lysosomal pathway (9). Cathepsins are important acidic endolysosomal proteases involved at different levels during the processes of the innate and adaptive immune responses. In the endocytic pathway, they are major players in direct pathogen killing, processing of human leukocyte antigen (HLA) class II molecules, antigen processing and presentation, proinflammatory signalling molecular turnover, and secretion of proinflammatory cytokines (10–15). While these cysteine proteases are optimally active in the acidic endolysosomal environment, they remain active in more neutral pH compartments (16, 17). They also operate i) in the cytosol, regulating apoptosis, pyroptosis, and inflammasome activation (18–20); ii) in the nucleus controlling gene expression (21); and iii) in the extracellular environment where they control extracellular matrix remodelling (22). Extracellular secreted cathepsins were found relevant for HIV transmission through genital mucosae (cathepsin D) (23) and for granuloma cavitation and lung parenchyma destruction during active TB (cathepsins K, G, and D) (24–26).

Not surprisingly, the abnormal activity of these proteases can lead to serious dysfunction and pathology and thus needs to be tightly controlled by endogenous protease inhibitors. Vital among endogenous protease inhibitors are cystatins (Csts), a group of evolutionarily related proteins. Under a normal physiological context, Csts control excessive cathepsin activity through trapping and blocking proteolytic activity in cells, extracellular milieu, organs, and body fluids. A slight imbalance in the equilibrium between Csts and cathepsins may result in unwanted inhibition of enzymatic activity (12). Type I Csts (also known as stefins) include CstA and B and are mainly found in the cytosol and the nucleus. In contrast, type II Csts are secreted and work as extracellular proteins, such as in the skin epithelia (Cst EM) and in saliva (Csts S, SA, SN, and D) [reviewed in (27, 28)]. Some secreted type II Csts, such as CstC and F, can be internalized by immune cells or translocated from the secretory pathway, thus accumulating in endosomal/lysosomal vesicles (29, 30). Type III Csts family members include kininogens circulating in the blood as precursors of the vasoactive peptide kinin [Cst families reviewed in (27, 28)].

Upon the classical activation by lipopolysaccharide (LPS) or by interferon-γ (IFN-γ), Mø redirect gene expression to upregulate a variety of proteases involved in direct killing of intracellular pathogens or indirectly by having a critical role in antigen processing and presentation (14, 15, 31). We previously demonstrated that a general downregulation of cathepsins including cathepsin S occurs either in resting or in IFN-γ-activated human Mø infected with Mtb (14, 15, 31). This may be a strategy used by this pathogen to manipulate the host microbicidal responses in order to survive intracellularly. In contrast, the infection with the non-pathogenic species *Mycobacterium smegmatis* led to a strong upregulation of most cathepsins in resting Mø, but a slightly weaker response was noted in activated Mø. Furthermore, with the exception of cathepsin F, we provided evidence that most cathepsins are involved in Mtb killing (14). Additionally, manipulation of cathepsin S expression led to improved intracellular killing of Mtb and increased MHC-II-antigen presentation and T-cell proliferation (15, 32).

In line with this mechanism, during *de novo* infection, HIV is able to counteract lysosome-mediated total degradation by markedly decreasing the expression of lysosomal cathepsins B, C, S, and X (33).

Our previous findings pointed out potential roles of protease inhibitors during Mtb infection. Indeed, treatment of Mtb-infected Mø with synthetic cathepsin inhibitors, such as E-64d, helped the bacteria to survive. Accordingly, internalization of exogenous CstC, the strongest inhibitor of cathepsins, led to a significant fivefold increase in Mtb survival rate 24 h after Mø infection (14). Since there have been no systematic studies in human primary Mø on the role of Csts during Mtb infection, especially during Mtb/HIV coinfection, we performed a transcriptomic analysis focusing on type I and II Csts. We found distinct gene expression profiles depending on Mø mono-infected with either Mtb or HIV or in Mø coinfected with both pathogens. In opposition to the profile found during infection with *M. smegmatis*, a species that is completely cleared in Mø in 24 to 48 h, we found CstC with the most prominent increased gene expression along that time in all tested pathogenic conditions. Csts C and F are the inhibitors described to accumulate in endocytic pathway (29, 30). Our results revealed CstF upregulated during *M. smegmatis* and Mtb infections but not during HIV or during Mtb/HIV coinfection. Altogether, the results suggest CstC as a major target working in the endolysosomal pathway during infection with all pathogens. Overall, our results propose the targeted modulation of CstC expression level in Mø as a potential therapeutic avenue to control Mtb infection including MDR-TB.

## Materials and Methods

### Cells and Culture Conditions

Primary human monocyte-derived Mø were obtained from buffy coats of healthy donors provided by the National Blood and Transplantation Institute (Instituto Português do Sangue e da Transplantação, Lisbon, Portugal) following a protocol established between Dr. Anes (Faculty of Pharmacy, University of Lisbon) and the blood institute. The personal details of the donors were not provided by the supplier. Briefly, peripheral blood mononuclear cells (PBMCs) were first isolated by density gradient centrifugation using Ficoll-Paque Plus (GE Healthcare). The PBMC fractions were incubated with anti-CD14 magnetic beads (Miltenyi Biotec), and the CD14^+^ monocytes were isolated using magnetic-activated cell separation (MACS) cell separation magnetic columns. Monocyte differentiation to Mø was induced by allowing them to adhere to 12-, 48-, or 96-well plates at 1 × 10^6^, 1.5 × 10^5^, or 5 × 10^4^ cells per well, respectively, for 2 h at 37°C, 5% CO_2_, in Roswell Park Memorial Institute (RPMI)-1640 medium (HyClone, GE Healthcare). Following adherence, the medium was supplemented to achieve a final concentration of 10% (v/v) fetal bovine serum (FBS) (HyClone, GE Healthcare), 1 mM of sodium pyruvate (HyClone, GE Healthcare), 10 mM of HEPES (HyClone, GE Healthcare), 0.1% β-mercaptoethanol (Gibco), and 20 ng/ml of the recombinant human M-CSF (BioLegend). The cell culture medium was renewed every 3 to 4 days until day 7 of differentiation. Purity (>99%) of the isolated culture was verified by flow cytometry (data not shown).

### Bacterial Cultures and HIV Isolates

*M. tuberculosis* H37Rv [American Type Culture Collection (ATCC) 27294], H37Rv GFP-expressing strain, *Mycobacterium bovis* BCG Pasteur (ATCC 35734), and the clinical strains isolated from patients with active TB were grown in Middlebrook’s 7H9 medium supplemented with 10% OADC enrichment (Difco), 0.02% glycerol, and 0.05% tyloxapol at 37°C (15). The strain *M. smegmatis* mc^2^155, containing a p19 (long lived) EGFP plasmid, was kindly provided by Dr. Douglas Young (The Francis Crick Institute, London, UK), and it was grown in medium containing Middlebrook’s 7H9 Medium (Difco) supplemented with 0.5% glucose and 0.05% tyloxapol at 37°C on a shaker at 200 rpm (34). The clinical strains were provided and characterized by the TB National Reference Laboratory from the Portuguese National Institute of Health Dr. Ricardo Jorge (INSA). The clinical strain (INSA code 33427) is susceptible to streptomycin, isoniazid, rifampicin, and pyrazinamide (PZA); and the MDR strain (INSA code 34192) is resistant to all those antibiotics plus ethionamide.

The primary isolate HIV-1_UCFL1032_ is part of our viral library stablished and maintained during the last three decades. This viral library contains a significant amount of HIV-1 and HIV-2 isolates characterized both genetically and phenotypically (35). HIV-1_UCFL1032_ was isolated from a seropositive individual after cocultivation of infected patient’s PBMCs with phytohemagglutinin (PHA)-stimulated PBMCs from uninfected individuals. Viral stocks were established in PBMCs from low-passaged supernatants of original cultures, aliquoted, and maintained at −80°C until used. Viral concentration was measured by reverse transcriptase (RT) activity using an ELISA (Lenti-RT kit, CavidiTech, Uppsala, Sweden). Phenotypic characterization made as described (35) on GHOST CD4+ cells individually expressing different coreceptors revealed that it uses CXCR4 coreceptor to enter host cells and has the ability to infect Mø producing low amounts of viral progeny upon inoculation, a phenotype similar to what is described during the course of Mø infection in patients (36). Phylogenetically, it belongs to subtype B. The usage of CXCR4 was confirmed by preincubating PBMC-derived Mø with AMD3100, an antagonist of CXCR4 as described previously (37–39). This was also confirmed by the absence of proviral DNA integration by nested PCR. The usage of CXCR4 has been referred as a possible viral entry route for Mø tropic HIV; and in HIV-1-infected individuals, it was shown that this coreceptor usage broadens as the disease progresses (38–41).

All experimental procedures using live Mtb and HIV were performed in the Biosafety Level 3 laboratory at the Faculty of Pharmacy of the University of Lisbon, respecting the national and European containment level 3 laboratory management and biosecurity standards, based on applicable EU Directives. All procedures have been approved by the faculty’s biological safety committee.

### Macrophage Infection

Prior to infection, bacterial cultures on exponential grown phase were centrifuged and washed in phosphate-buffered saline (PBS) and then resuspended in Mø culture medium without antibiotics. Bacterial clumps in the suspension were dismantled by ultrasonic bath treatment for 5 min. The suspension was further centrifuged for 1 min at 500 × *g* to remove residual clumps. Single-cell suspension was verified by fluorescence microscopy and quantified by optical density at 600 nm.

The infection was performed with a multiplicity of infection (MOI) of 1 bacterium per Mø and with the equivalent of 1 ng of RT of HIV-1_UCFL1063_ per ml. After 3 h of infection at 37°C, 5% CO_2_, the cells were washed with PBS to remove free bacteria/virus and cultivated in fresh complete medium.

Phagocytosis of the bacteria was evaluated by flow cytometry using *M. tuberculosis* H37Rv GFP-expressing strain and following the procedures described below. Monitoring of HIV infection was performed by fluorescence microscopy. Infected Mø were fixed with 4% paraformaldehyde–4% sucrose solution in PBS for 1 h and quenched with 50 mM of NH_4_Cl in PBS for 15 min. Cells were permeabilized with 0.1% Triton X-100 for 5 min and blocked with 1% bovine serum albumin (BSA) in PBS for 30 min. Cells were stained with anti-Gag antibody diluted 1:100 (KC57, Beckman Coulter) in 1% BSA for 1 h, washed, and then incubated with Alexa Fluor 555 Goat anti-Mouse IgG secondary antibody (dilution of 1:1,000; Cell Signaling Technology) for 30 min. Coverslips were mounted using ProLong Gold Antifade Mountant (Thermo Fisher Scientific) and visualized on a Leica TCS SP8 confocal microscope. Non-infected cells were used in parallel as a negative control for the specificity of anti-Gag antibody labelling. Analysis was performed using Leica Application Suite X and ImageJ software. To further confirm the integration of the viral DNA into the host genome, a nested PCR was performed as previously described (42). Briefly, the first round of PCR amplification was performed using an *Alu*-specific sense primer in combination with a *gag* antisense HIV-1 specific primer; the PCR products were then subjected to a second amplification reaction targeting the HIV-1 R/U5 region of long terminal repeat (LTR).

### Flow Cytometry

Following 24 h of infection, Mø in 48-well plates were recovered with HyQTase cell detachment solution (HyClone, GE Healthcare). For the identification of apoptotic and necrotic cells, Annexin V-FITC Kit (Miltenyi Biotec) was used following the manufacturer’s instructions. Cells were incubated with annexin V and propidium iodide for 20 min, washed with the appropriate kit buffer, fixed in 4% paraformaldehyde solution, and prepared using the same buffer, for 1 h. Following fixation, cells were washed again in buffer and analyzed. For surface staining of HLA molecules, detached cells were promptly fixated for 1 h. Following fixation, cells were washed and incubated with Human TruStain FcX Fc receptor blocking solution (BioLegend) for 10 min and then stained for 20 min with antibodies specific for human HLA class I (Cat # 311422, BioLegend) and HLA class II (Cat # 361716, BioLegend) molecules. Samples were analyzed in Guava easyCyte™ 5HT flow cytometer.

### Reverse Transcriptase–qPCR

Immediately following a 24- or 48-h infection, RNA was isolated from Mø in 12-well plates. RNA isolation was performed using NZY Total RNA Isolation kit (NZYTech), following the manufacturer’s instructions. Total RNA measuring 200 ng was used for cDNA synthesis with NZY First-Strand cDNA Synthesis Kit (NZYTech), according to the manufacturer’s instructions. qPCR was performed using NZY qPCR Green Master Mix (NZYTech) with the different sets of primers (**Table 1**) (Eurofins Genomics) at a final concentration of 0.5 μM.

**Table 1:** List of qPCR primers.

Target gene		Target sequence (5′–3′)
Cystatin A	Forward	AAACCCGCCACTCCAGAAAT
	Reverse	TTATCACCTGCTCGTACCTTAAT
Cystatin B	Forward	TGTCATTCAAGAGCCAGGTG
	Reverse	AGCTCATCATGCTTGGCTTT
Cystatin C	Forward	CAACAAAGCCAGCAACGACAT
	Reverse	AGAGCAGAATGCTTTCCTTTTCAGA
Cystatin D	Forward	GATGAGTACTACAGCCGCCC
	Reverse	AGCAGAACTCTTCCTCTTTCAGT
Cystatin E/M	Forward	TCCGAGACACGCACATCATC
	Reverse	TCACAGCGCAGCTTCTCCT
Cystatin F	Forward	TCCCCAGATACTTGTTCCCAGG
	Reverse	TTCTGCCAATTTCCACCTCCA
Cystatin S	Forward	GCTCCAGCTTTGTGCTCTGCCT
	Reverse	GTCTGCTCCCTGGCTCGCAG
Cystatin SA	Forward	CTGCGGGTGCTACGAGCCAG
	Reverse	GGAGGGAGGGCAGAGTCCCC
Cystatin SN	Forward	TCCCTGCCTCGGGCTCTCAC
	Reverse	ACCCGCAGCGGACGTCTGTA
GAPDH	Forward	AAGGTGAAGGTCGGAGTCAA
	Reverse	AATGAAGGGGTCATTGATGG

The PCR proceeded as follows: 1 cycle of 95°C for 10 min, followed by 40 cycles of 95°C for 15 s, 60°C for 30 s, and 72°C for 30 s. The qPCR was performed using a QuantStudio™ 7 Flex System (Thermo Fisher). The data were analyzed using the ΔΔCt method in the Applied Biosystems™ Analysis Software. The mRNA expression profiles were normalized with respect to glyceraldehyde 3-phosphate dehydrogenase (GAPDH) housekeeping gene and finally calculated relative to non-infected samples. For each condition, three biological replicates were tested; and for every biological replicate, two technical replicates were performed. Statistical analysis was performed by ANOVA two-parameter. Gene expression heatmaps were generated using TM4 MultiExperiment Viewer software.

### Transfection

Transfection with anti-Cst C siRNA or with scramble control siRNA was performed with ScreenFect A (ScreenFect) transfection reagent and following the manufacturer’s protocol. Mø were incubated for 24 h with the transfection reagent and 50 nM of SMARTpool ON-TARGETplus human CST3 siRNA (Dharmacon, USA; target sequences: CAAUGACCUUGUCGAAAUC, CGUCGGCGAGUACAACAAA, GAACCACGUGUACCAAGAC, and UAGCUAGGGGUGAACUACUU) or the respective siRNA non-targeting control (Dharmacon, USA; target sequences: UGGUUUACAUGUCGACUAA, UGGUUUACAUGUUGUGUGA, UGGUUUACAUGUUUUCUGA, and UGGUUUACAUGUUUUCCUA) in antibiotic-free medium. Following this incubation, fresh medium was added, and the cells were incubated for an additional 2 days prior to any experiment in order to achieve maximum silencing. Silencing efficacy was measured by qPCR and Western blotting.

### Western Blotting

Total proteins were harvested using Laemmli buffer (Sigma-Aldrich) and heated at 95°C for 5 min. Samples were subjected to sodium dodecyl sulfate–polyacrylamide gel electrophoresis (SDS-PAGE) in 15% polyacrylamide gel and transferred to 0.2-μm pore nitrocellulose membrane (Amersham Protran, Cytiva). Membrane was blocked in 5% low-fat milk PBS with 0.1% Tween 20. Following blocking, the membrane was incubated in 1:2,000 dilution of primary antibodies specific for CstC (Cat # ABC20, Sigma-Aldrich) and β-tubulin (Cat # ab6046, Abcam) overnight. Membranes were washed in PBS-Tween and incubated with secondary horseradish peroxidase (HRP)-conjugated antibody (Cat # 1706515, Bio-Rad) for 1 h. Bands were visualized by chemiluminescence using NZY Supreme ECL HRP substrate (Cat # MB19301, NZYTech) in a ChemiDoc XRS (Bio-Rad). Quantification of band intensity was performed on ImageJ software.

### Colony-Forming Unit Assay

When required, infected Mø in 96-well plates were lysed in 0.05% Igepal solution. Serial dilutions of the resulting bacterial suspension were plated in Middlebrook’s 7H10 with 10% OADC (Difco) and incubated for 2–3 weeks at 37°C before colonies were observable.

### Enzymatic Activity of Cathepsins

Following 24 h of infection, Mø in a 96-well plate were washed with PBS and incubated in PBS with OmniCathepsin (Z-FR-AMC, Z-Phe-Arg-AMC) (Enzo Life Sciences) or cathepsin S (Z-VVR-AFC) (BioVision) fluorogenic substrate at 37°C in a Tecan M200 spectrofluorometer. Fluorescence readings were performed every 5 min. Assay specificity was verified by treating the cell lysates with general protease inhibitor E-64d or with specific cathepsin S inhibitor provided in the kit.

### CD4^+^ Lymphocyte Proliferation

Autologous CD4**^+^** lymphocytes were obtained from healthy PPD^+^ donors according to the isolation protocol described above. Positive selection of the CD4**^+^** lymphocytes was performed using anti-CD4 magnetic beads (Miltenyi Biotec). Isolated lymphocytes were cultivated in 75-cm^2^ flask at 2 × 10^6^ cells per ml in RPMI-1640 medium (HyClone, GE Healthcare) supplemented with 15% (v/v) FBS (HyClone, GE Healthcare), 1 mM of sodium pyruvate (HyClone, GE Healthcare), 10 mM of HEPES (HyClone, GE Healthcare), and 20 UI/ml of human recombinant interleukin-2 (BioLegend) for 3 days prior to the experiment. Immediately before the experiment, the lymphocytes were stained with carboxyfluorescein diacetate succinimidyl ester (Cat # 423801, BioLegend) following the manufacturer’s instructions. Mø infected with *M. tuberculosis* H37Rv or *M. bovis* BCG for 24 h were washed and cocultivated with the lymphocytes at a ratio of five lymphocytes per Mø for 5 days. CD4**^+^** lymphocytes were recovered after 5 days of coculture and analyzed using Guava easyCyte™ 5HT flow cytometer.

### Sandwich ELISA for IFN-γ Quantification

Supernatants from the previous assay were recovered following 5 days of coculture with CD4**^+^** lymphocytes and stored at −80°C for further analysis of IFN-γ secretion. The quantification was performed by sandwich ELISA using ELISA Max Deluxe Set Human for IFN-γ (Cat # 430104, BioLegend) kit and following the manufacturer’s instructions. Absorbance was measured by Tecan M200 spectrofluorometer at 450 and 570 nm.

### Statistical Analysis

Statistical analysis was performed using SigmaPlot 12. Multiple group comparisons at different time points of qPCR data were performed using ANOVA two-parameter test followed by pairwise comparisons of the groups using the Holm–Sidak test. Multiple group comparisons of the rate of cathepsins’ proteolytic activity were made using one-parameter ANOVA followed by pairwise comparisons of the groups using the Holm–Sidak test. Two group comparisons of gene silencing efficacy, bacteria colony-forming unit (CFU), Mø HLA expression, lymphocyte proliferation, and IFNγ secretion between scramble control and CstC siRNA were made using Student’s t-test. All the prerequisites of the tests were verified. The considered nominal alpha criterion level was 0.05, below which differences between samples were deemed significant.

## Results

### Low Multiplicity of Infection of Human Macrophages With *Mycobacterium tuberculosis* Combined With High HIV Viral Inoculum Does Not Impact Cell Death

**Figure 1:**
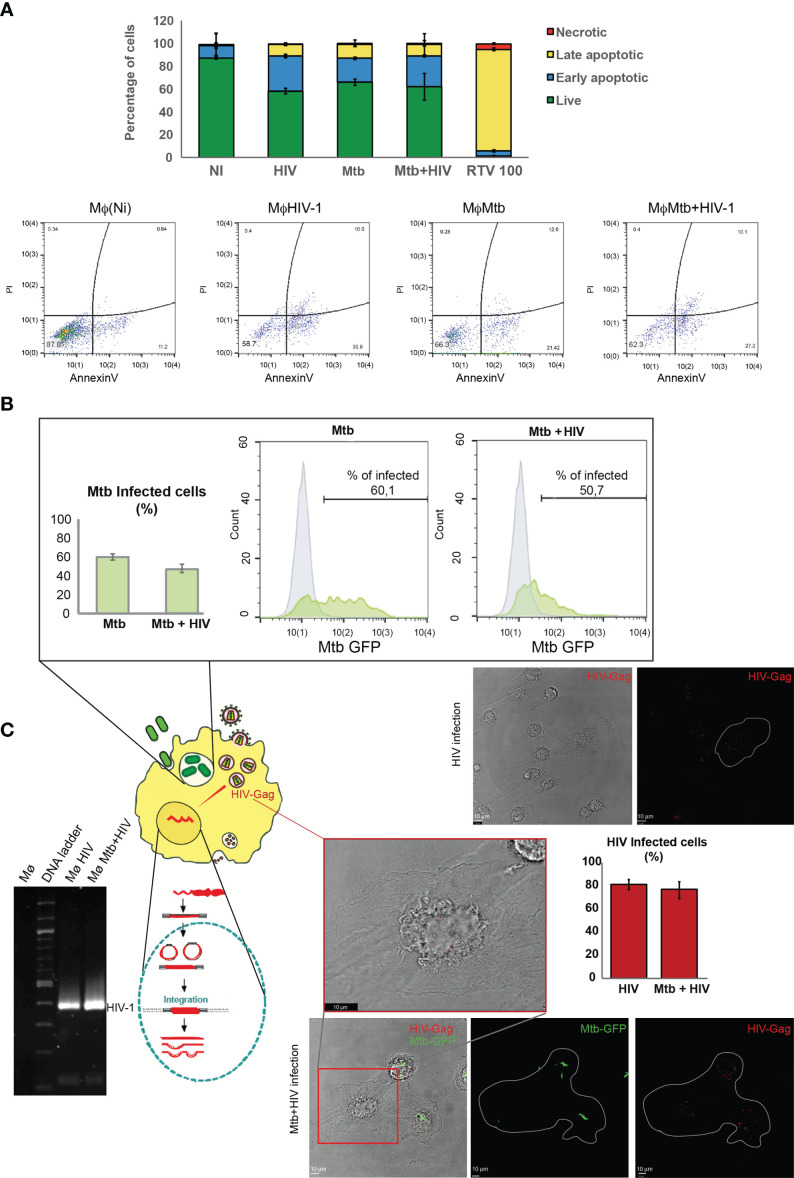
Infection with either Mtb or HIV, or coinfection with pathogens, in human Mø does not impact cell death. **(A)** Flow cytometry analysis of the percentage of infected cells stained for annexin V and/or with propidium iodide 48 h p.i. Results represent the mean of biological triplicates for each challenge. **(B)** Percentage of Mtb-infected Mø during mono-infection or during coinfection with HIV after 3 h p.i. determined by flow cytometry. Bar plot depicts the mean ± SEM of three independent experiments. **(C)** HIV infection of Mø during mono- or coinfection with Mtb. Images represent z-axis maximum intensity projections of Gag/p24 protein depicted in red and Mtb depicted in green on a single plane of the bright-field channel, visualized by confocal microscopy. Bar plots represent the mean percentage of cells infected by HIV obtained from the microscopic analysis of 250 cells per treatment in ImageJ software. Error bars show the standard deviation. Gel electrophoresis shows the result of nested PCR amplification of HIV-1 (92US660) LTR with 391 base pairs (bp). Mtb, *Mycobacterium tuberculosis*; Mø, macrophages; p.i., post infection; LTR, long terminal repeat.

It is well established that HIV impairs the ability to control Mtb infection and vice versa (7, 8, 43). To standardize the infection of Mø in order to maintain similar numbers of viable cells during infection with either Mtb or HIV, or coinfection with both pathogens, we established different combinations of bacteria MOI and the viral infecting inoculum. Our previous results indicated that a MOI of up to 1 for Mtb in Mø derived from peripheral blood monocytes does not significantly impact cell death in our experimental conditions and time points analyzed (44). Therefore, we investigated a standard bacteria MOI of 1 versus distinct viral inocula produced by serial dilution assays. The best ratio combination to observe a high percentage of infected cells with similar cell death was 1 ng of HIV-1 RT per 10^6^ cells per ml. Cell death was evaluated by flow cytometry using annexin V to stain apoptotic cells and propidium iodide for necrotic cells at 24 and 48 h post infection (p.i.). As shown in **Figure 1A**, apoptosis was much more prominent than necrosis in all conditions tested, but with a similar percentage when comparing HIV and Mtb mono-infection with coinfection. Moreover, the total amount of Mø was similar between the distinct conditions, and no cell population changes in size and granularity that could interfere in the analysis were detected (**Figure S1**). Thus, we proceeded with MOI of 1 for Mtb and 1 ng of HIV for all subsequent infections. Ritonavir, a protease inhibitor formerly used for HIV therapeutics, was used in toxic concentrations as positive control (32).

In an attempt to decipher if the simultaneous infection with both pathogens differentially interferes with internalization relative to mono-infections in our *in vitro* model, we quantified bacterial internalization by flow cytometry analysis of Mø infected with the Mtb-GFP strain. As expected, approximately 60% of all Mø were infected at 3 h p.i., with Mtb (**Figure 1B**). In experimental conditions, coinfection with HIV of up to 10% decrease in the internalization of Mtb was observed (**Figure 1B**). Viral internalization was evaluated by immunofluorescence labelling of the Gag protein in infected cells as shown in **Figure 1C**. Quantification of infected cells was done 3 h p.i. using a parallel culture of HIV-infected Mø exposed to the exact same conditions as the one used for coinfection with Mtb. No signal was detected in non-infected cells, confirming that the red fluorescence detected in the cytosol corresponds to virus-encoded Gag protein. ImageJ software analysis of the images showed that about 80% of cells expose to HIV particles internalized the virus. Most cells displayed small numbers of dots in the cytoplasm. A large number of cells tend to concentrate them at the nuclear region. This finding is in accordance with previous results showing viral capsid concentration in nuclear region before retrotranscription (45) and with viral staining of Gag visualized by confocal microscopy 6 h p.i in cytosol and in perinuclear region of Mø (46). Since one of the steps of virus replication cycle is the integration of the retroviral DNA into the host genome, a nested PCR (**Figure 1C**) revealed an amplicon of 391 bp, thus further confirming HIV infection of Mø.

Altogether, these results demonstrate an optimization of our mono- and coinfection models to study the modulation of the desired gene expression profile.

### Cystatin Expression Is Differentially Regulated in Macrophages During Infection With Either *Mycobacterium tuberculosis* or HIV, and Coinfection With Both Pathogens

We next aimed to assess the pattern of type I and II Cst mRNA gene expression during our infection conditions in Mø. We performed a qRT-PCR analysis of type I and II Csts expressed in Mø at early stages of infection (24 and 48 h p.i.). This rationale was based on previous gene expression screens where it was shown that the majority of the host’s genes are modulated during the first 24–48 h p.i. with Mtb (47).

**Figure 2:**
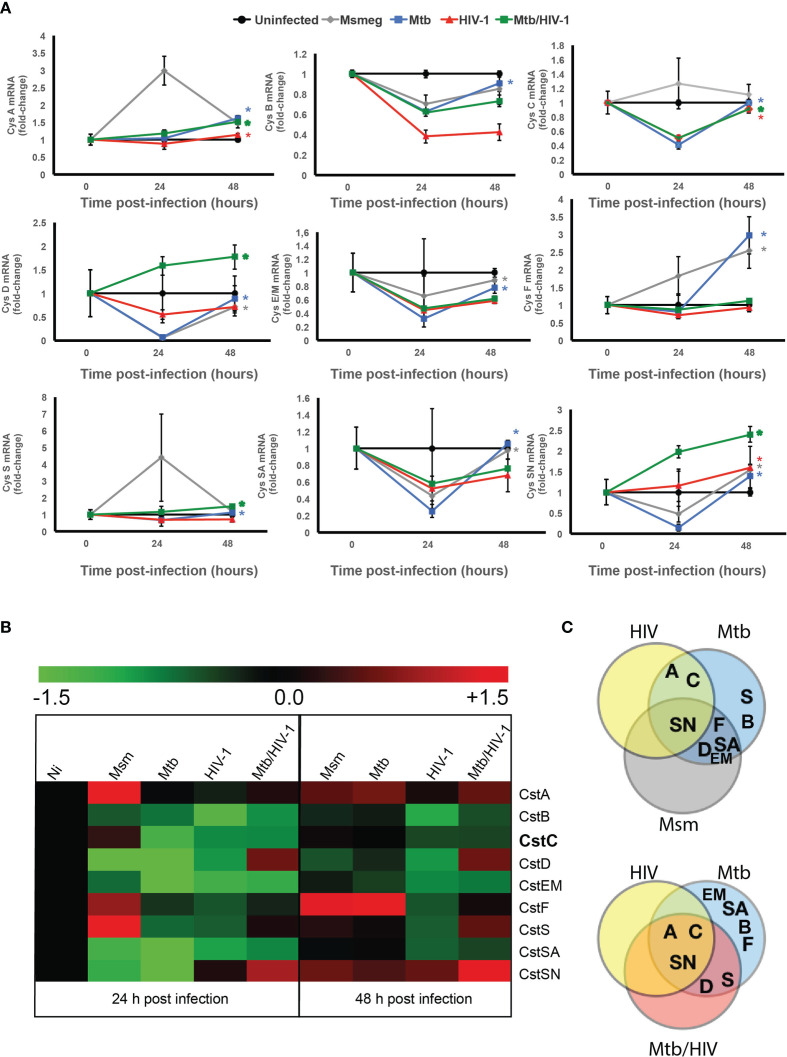
Cystatins are differentially regulated during mono-infection with Mtb or HIV, or coinfection with pathogens. **(A)** Gene expression kinetics of cystatins in Mø infected with Mtb or HIV, or coinfected with both pathogens, in comparison with the infection with *Mycobacterium smegmatis* for 24 and 48 h. Values are depicted relative to uninfected control and were previously normalized to GAPDH expression. Data are represented as the mean fold change per sample ± standard error. Statistical significance displayed refers to the values at 48 h relative to 24 h p.i. (*p ≤ 0.01; n = 3). **(B)** Heatmap of qRT-PCR quantification of mRNA obtained from Mø after 24 and 48 h of infection. Values are depicted as log2 gene expression relative to uninfected Mø. **(C)** Venn diagram of the confirmed “hits” that exhibit significantly increased gene expression from 24 to 48 h p.i. Mtb, *Mycobacterium tuberculosis*; Mø, macrophages.

In addition, we infected Mø with *M. smegmatis* to assess gene expression differences in response to a non-pathogenic mycobacteria, which is usually cleared within Mø 24–48 h after challenge (14, 48). As shown in **Figures 2A, B**, CstB, a type I Cst, was downregulated at 24 and 48 h p.i., independently of the type of microorganism challenge. By contrast, CstA, the other Cst of the same family, displayed an increasing expression tendency in response to pathogen challenge, including a prominent upregulation in response to *M. smegmatis* at 24 h p.i. (**Figures 2A, B**). For the other tested Csts belonging to the group II family, we observed a general downregulation tendency upon pathogen challenge with few exceptions. In the case of the *M. smegmatis* challenge, a species that is almost completely cleared in the first 24 h p.i (48)., we observed the upregulation of CstC, CstF, and CstS. For Mtb, there was an upregulation of CstF and CstSN; for HIV, there was an upregulation of CstSN; and for Mtb and HIV coinfection, CstD, S, and SN were all strongly upregulated 48 h p.i. (**Figures 2A, B**).

As stated before, a slight imbalance in the equilibrium between Csts and cathepsins may result in unwanted inhibition of enzymatic activity (12). This is particularly relevant for strong inhibitors of cathepsins such as CstC or F, contrary to weak inhibitors such as CstD, S, and SN, where a slight variance in protein concentration may induce a major switch from non-activity to strong enzymatic inhibition. We then compared the evolution of gene expression between the time points 24 and 48 h. In order to have an overview of this Cst differential gene expression, we performed a Venn diagram analysis (**Figure 2C**). In the Venn figure, CstSN (at the top) was unique in that it increases gene expression from 24 to 48 h p.i. with all microorganisms. Regarding the mono-infection data, we noticed that CstA and CstC are differently modulated during infection either with Mtb or HIV in comparison with *M. smegmatis* (**Figure 2C**, top panel). When we compared the mono-infections with Mtb or HIV to the coinfection context, we confirmed that CstA and CstC are uniquely modulated by these pathogens (**Figure 2C**, bottom panel) and with statistically significant increased gene expression between those time points. Moreover, the analysis of Csts basal expression in non-infected cells (**Figure S2**) shows that Csts A, B, and C possess a high level of basal expression comparable with the housekeeping gene GAPDH, while the remaining Csts are 100- to 10,000-fold less expressed. Taking this into consideration, slight variations in gene expression for Csts A and C may result in more drastic effects on cathepsin activity.

These findings indicate that CstA and CstC constitute obvious candidates for further evaluation in our infection model.

### Inactivation of CstC Expression in Primary Human Macrophages Results in Increased *Mycobacterium tuberculosis* Killing During Mono- and Coinfection With HIV

**Figure 3:**
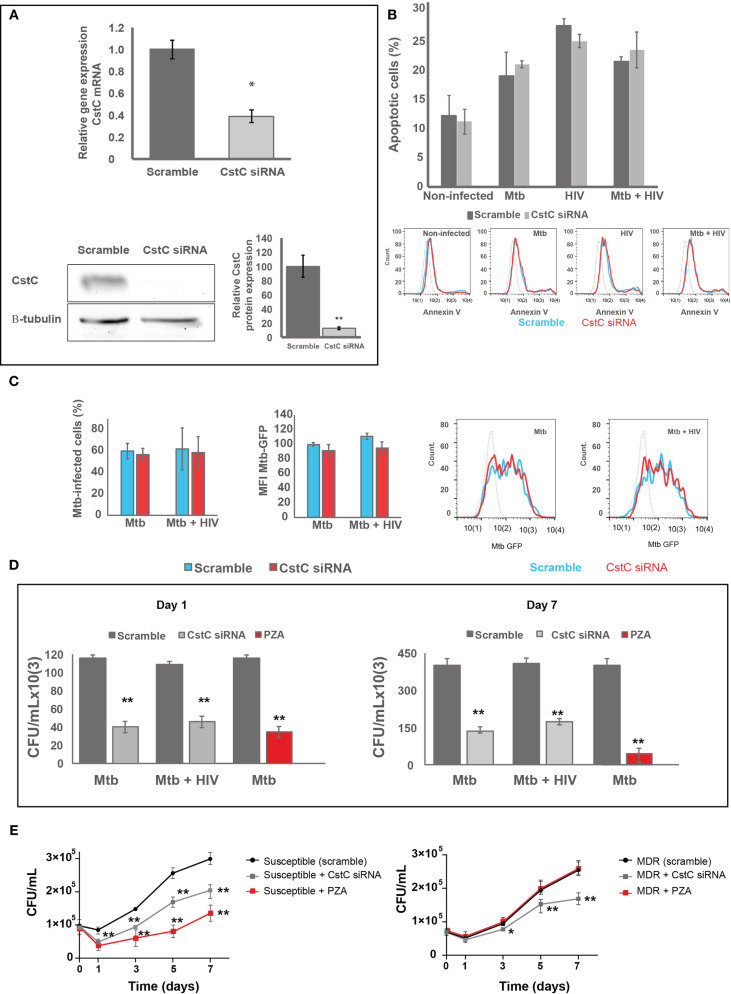
SiRNA-mediated gene silencing for CstC results in increased Mtb killing in primary human Mø during mono-infection and during HIV coinfection. **(A)** CstC was silenced by siRNA 3 days prior to infection in order to achieve maximum protein silencing. Relative gene expression of CstC mRNA in Mø was determined by RT-qPCR and Western blotting at 24 h p.i. Values are relative to cells transfected with scramble and represent the average of biological triplicates (*p < 0.01; n = 3). **(B)** Effects of siRNA for CstC relative to scramble transfected cells on apoptosis in infected Mø. Apoptosis was measured by flow cytometry after 24 h of infection using fluorescent annexin V antibodies. Values show median fluorescence intensity (MFI) from one representative experiment performed in triplicate, while error bars depict the standard deviation. **(C)** Percentage of Mø infected with Mtb, and MFI of Mtb per Mø were measured by flow cytometry in scramble infected cells and in CstC-silenced infected cells, after 3 h of infection with a GFP-expressing Mtb strain. Bar plots depict the average of three biological replicates, and the error bars depict the standard error. Raw values from one representative replicate are presented in the fluorescence intensity histograms. **(D, E)** Intracellular survival of Mtb: reference laboratory strain H37Rv **(D)** and clinical strains **(E)**. Colony-forming units (CFU) of intracellular bacteria were recovered from Mø transfected with siRNA for CstC or with a scramble siRNA. Values depict mean CFU representative of three biological replicates measured in duplicate, while the error bars depict the SD. Asterisks indicate statistical significance between samples at the same time point relative to scramble control (*p < 0.01; **p < 0.001; n = 3). PZA was used as control for killing efficacy. Mtb, *Mycobacterium tuberculosis*; Mø, macrophages; PZA, pyrazinamide.

CstC is described as the most prominent Cst in immune cells and one of the few that accumulate in endolysosomal vesicles, with particular strong inhibitory effects on cathepsins such as B, L, and S (29, 30). In fact, our previous studies revealed that exogenous supplementation with CstC helped Mtb survival in human monocyte-derived Mø (14). Therefore, we decided to further investigate the downregulation of CstC expression during mono-infection with Mtb and coinfection with HIV, as potential effect by host Mø to counteract Mtb intracellular colonization. To this end, we performed siRNA-mediated gene silencing to decrease specifically the expression of CstC, as previously established in primary human Mø (49). As shown in **Figure 3A**, approximately 60% silencing of CstC mRNA was achieved, and that translated into a strong reduction of CstC protein level (**Figure 3A** and **Figure S3**). Importantly, no difference in cell death was observed between CstC-silenced Mø compared with scramble controls (cells transfected with a non-specific RNA; see *Materials and Methods*) (**Figures 3B** and **S4**). Next, we compared the effect of CstC silencing on the internalization and intracellular survival of Mtb in Mø during mono- and coinfection with HIV. Flow cytometry analysis showed that approximately 60% of Mø were infected with similar amounts of bacteria when comparing CstC-silenced Mø with scramble controls (**Figure 3C**). Next, bacterial survival was assessed by CFU counts of bacilli recovered from infected cells. As such, we observed a strong and significant reduction in CFU from bacteria recovered from CstC-silenced infected Mø relative to scramble controls (*p* < 0.001) (**Figure 3D**). At 24 h p.i., CstC silencing yielded around 70% increase in Mtb intracellular killing during mono-infection and 60% during coinfection with HIV. Interestingly, the impact CstC silencing on intracellular Mtb killing was similar to that obtained with PZA treatment (at a MIC of 100 μg/ml), a first-line antibiotic for TB. The effects on mycobacteria killing were maintained for up to 7 days p.i. We then proceeded to analyze the effects of CstC depletion on the intracellular survival of clinical strains isolated from TB patients (**Figure 3E**). For the susceptible strain, the results were similar to those obtained with the reference laboratory strain H37RV. For the MDR strain, we found a significant killing effect induced by CstC depletion, a strain for which the first-line antibiotics isoniazid, rifampicin, and PZA, plus ethionamide, have lost their efficacy.

Overall, our results reveal that the modulation of CstC expression in Mø is important to control infection.

### Inactivation of CstC Expression Impacts Cysteine Cathepsin Enzymatic Activity in Macrophages Infected With Either *Mycobacterium tuberculosis* or HIV, or Coinfected With Both Pathogens

**Figure 4:**
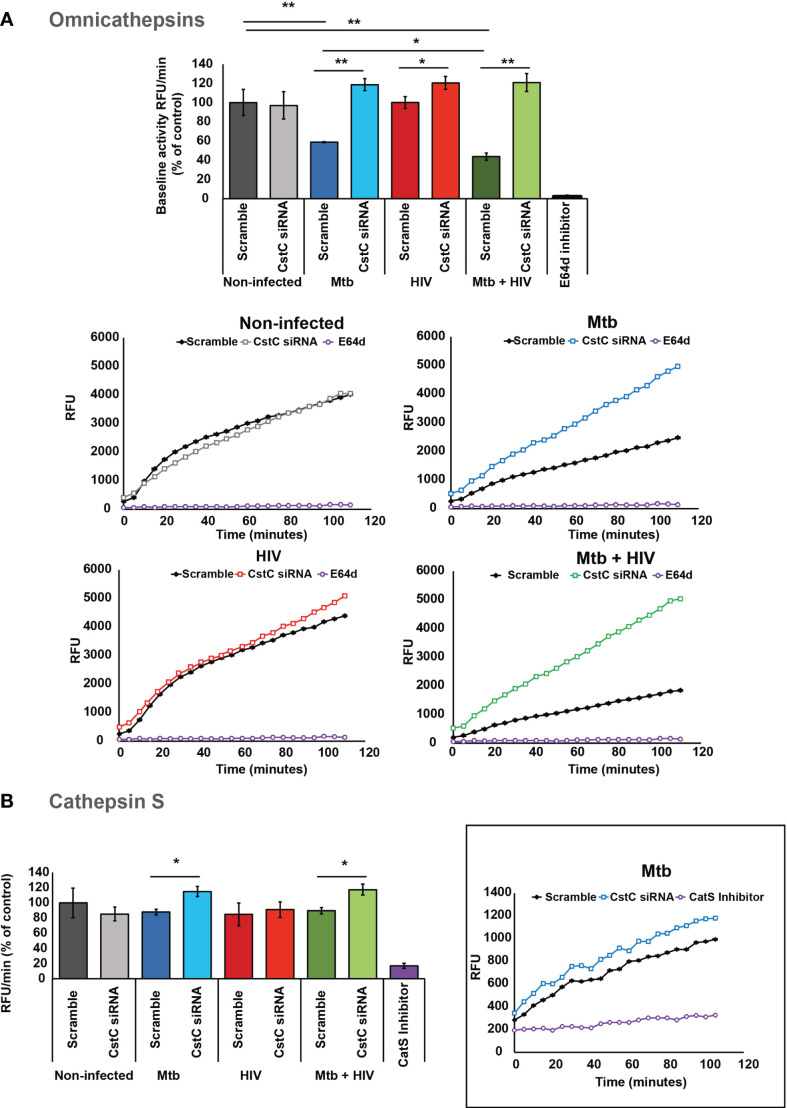
SiRNA-mediated gene silencing for CstC alter cathepsin activity in human Mø infected with Mtb. **(A)** OmniCathepsin or **(B)** cathepsin S activity alone was monitored with a specific fluorogenic substrate every 5 min in live cells (scramble-control and CstC-silenced cells), or with specific inhibitors (E-64d or ZFL-COCHOO for cathepsin S). The slope of fluorescence emission in the scramble control of non-infected cells was represented as 100%, and the effect of each sample was calculated as a percentage relative to control. Data are represented as average from three independent experiments, and the error bars represent standard error (*p < 0.01, **p < 0.001; n = 3). Mtb, *Mycobacterium tuberculosis*; Mø, macrophages.

To confirm if the impact of CstC silencing on the intracellular killing of Mtb was attributed to a direct effect on cathepsins, we assessed the OmniCathepsin proteolytic activities, which measures the combined activities of cathepsins B, L, and S. The cleavage of a peptidase-specific fluorogenic peptide substrate was measured over almost 2 h starting at 24 h p.i. The specificity of substrate cleavage was checked by preincubation of cells with E-64d, a cognate inhibitor of most cathepsins. As expected, Mtb infection induces a decrease in OmniCathepsin activity as shown by comparing the activity in scramble non-infected cells with Mtb scramble, or with scramble in coinfection. In all infection settings, CstC silencing leads to a significant increase in OmniCathepsin activity, with a twofold increase in the case of Mtb mono-infection (**Figure 4A**). In marked contrast, the silencing of CstC has no effect in cathepsin activities in non-infected cells, suggesting that the capacity of Mtb infection to lower cathepsin activity depends on CstC expression (**Figure 4A**). When compared with non-infected cells, mono-infection with HIV did not affect cathepsin activity (**Figure 4A**). However, the silencing of CstC led to a significant increase on OmniCathepsin activity of 0.2-fold during HIV infection (**Figure 4A**). Altogether, these results indicate that silencing of CstC impacts cathepsin B, L, and S activities in the endocytic pathway during Mtb and HIV mono-infections and during coinfection.

Due to the cathepsin S direct killing effect on intracellular pathogens, and its important role in adaptive immunity, we then followed the kinetics of cathepsin S activity. To do so, a cathepsin S-specific fluorogenic peptide substrate was employed along with a cathepsin S-specific inhibitor (see *Materials and Methods*) (15, 29, 50). Accordingly, we show that by silencing CstC, a slight, but significant, increase in cathepsin S activity was observed during Mtb infection and coinfection with HIV (**Figure 4B**). CstC depletion did not impact cathepsin S activity in mono-infection with HIV (**Figure 4B**), suggesting that, in the case of HIV infection, cathepsin B and/or L activity should be modified.

### CstC Depletion Increases the Cell-Surface Expression of Human Leukocyte Antigen Class II and CD4^+^ T-Lymphocyte Proliferation Along With IFN-γ Secretion

**Figure 5:**
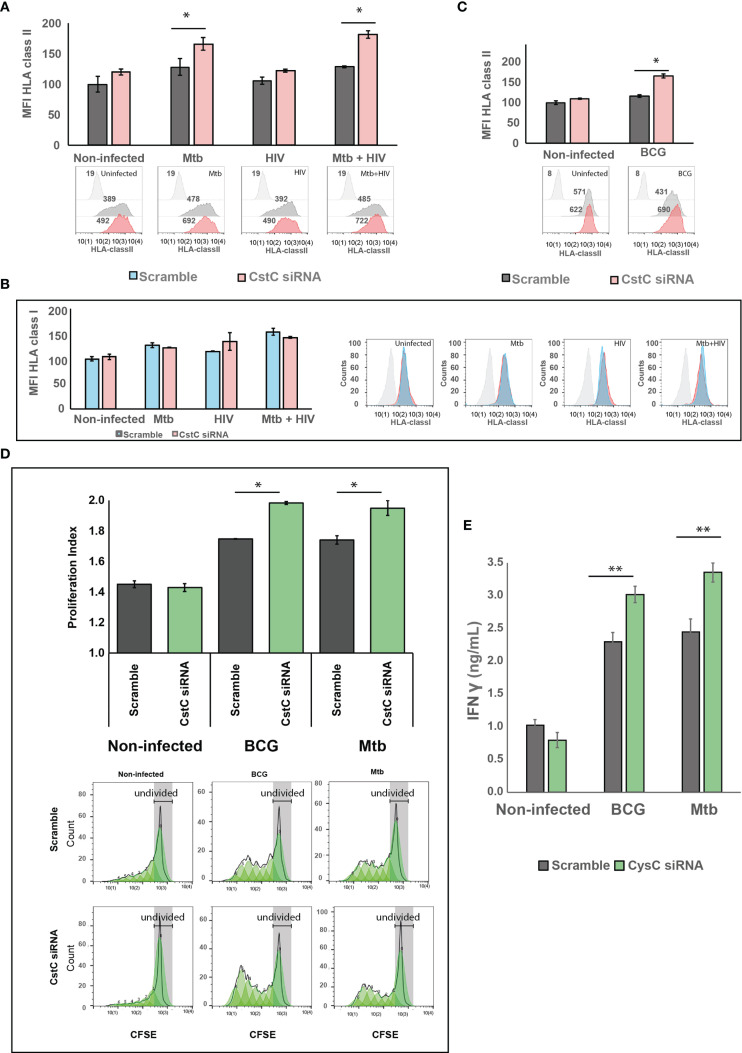
SiRNA-mediated gene silencing for CstC results in increased cell-surface expression of MHC class II and elevated T-cell proliferation and proinflammatory IFN-γ secretion. Cell-surface expression of human leukocyte antigen (HLA) class II **(A, B)** or class I **(C)** on Mø transfected with siRNA for CstC or with a scramble siRNA in the different infection challenges compared with non-infected cells. HLA class II and I were measured by flow cytometry after 24 h of infection. Values in bar plots represent median fluorescence intensity (MFI) relative to the respective scramble controls from one representative experiment performed in triplicate, while error bars depict the standard deviation (*p < 0.01; n = 3). **(D)** CD4^+^ T-cell proliferation after 5 days of coculture with Mtb- or BCG-infected Mø. Infected Mø were cocultivated with CFSE-stained CD4^+^ T cells following 24 h of infection. After 5 days of coculture, CD4^+^ T-cell CFSE fluorescence was measured by flow cytometry. Values in bar plots represent the proliferation index (average number of divisions per cell) of CD4^+^ T cell (*p < 0.01, relative to scramble control; n = 3). Histograms from one representative replicate of the different treatments infected with Mtb are presented at the bottom. The green areas represent the CD4^+^ T cell populations after each division, as modeled by the software. Each generation is identified by a 50% decrease in fluorescence caused by cell division. **(E)** IFN-γ was quantified in the supernatant after 5 days of cocultures of Mø with CD4^+^ T cells by ELISA. Values depict mean concentration of three biological replicates from one representative experiment performed in duplicate. Error bars depict the standard deviation (**p < 0.001, relative to control; n = 3). Mtb, *Mycobacterium tuberculosis*; Mø, macrophages; CFSE, carboxyfluorescein succinimidyl ester.

CstC has been implicated in the impairment MHC class II processing and in endosomal antigen processing and presentation by regulating the activity of cathepsin S (29, 50). We hypothesized that the noticeable Mtb-induced increase in CstC expression might be linked to poor antigen processing and presentation, thereby compromising the adaptive immunity response to infection. To test this, we silenced CstC in non-infected cells, Mtb- or HIV-infected cells, or coinfected with both pathogens, and then we assessed the surface expression of HLA class II molecules by flow cytometry. In all conditions with Mtb infection, we found an increased cell surface of HLA class II expression (**Figure 5A**). Mono-infection with HIV failed to accomplish this effect, indicating that Mtb is responsible for this result in in coinfected cells (**Figure 5A**).

Cathepsin S is also involved in partial antigen processing for cross-presentation to CD8^+^ T lymphocytes (51), but with no effects on HLA class I expression at the cell membrane (52, 53). We thus analyzed the expression of HLA class I at the cell surface by flow cytometry. No change was observed when comparing siRNA for CstC-treated cells relatively with scramble (**Figure 5B**).

We then focused on BCG infection. Since its first use in 1921, the BCG vaccine strain has lost its immunogenicity capacity. We decided to test if silencing of CstC results in improved surface expression of HLA class II molecules is required for antigen presentation, in the context of Mø infection with BCG. In a similar manner than for Mtb infection, the reduction of CstC gene expression significantly increases the cell surface of the MHC II class molecules in BCG-infected cells relative to scramble control infected cells (**Figure 5C**).

Finally, we questioned if the increase on cell-surface expression of MHC class II induced by CstC silencing would impact CD4^+^ T-lymphocyte proliferation. To this end, we performed cocultures of infected Mø with autologous CD4**^+^** T lymphocytes obtained from the same healthy PPD^+^ donors and evaluated their ability to induce T-cell proliferation (**Figure 5D**). Following the same pattern of HLA class II surface expression, CstC depletion in Mtb- or BCG-infected cells induced a significant increase of T-cell proliferation relative to scramble controls, after 5 days post-cocultivation as evaluated by flow cytometry (**Figure 5D**). As a consequence, we also observed an increased secretion of IFN-γ in coculture supernatants of Mtb- or BCG-infected cells, which is further enhanced in CstC-silenced cells (**Figure 5E**); no significant alterations in IFN-γ secretion were detected in non-infected cocultures.

Collectively, our findings demonstrate that the modulation of CstC expression in human Mø has significant consequences to the innate immune control of Mtb intracellular growth, which is later amplified in the capacity of these cells to activate the adaptive immune response probably through a defective processing and presenting of antigen *via* MHC class II.

## Discussion

Cathepsins were first described as endolysosomal proteases involved in the elimination of microorganisms or cell debris especially by professional phagocytic cells such as Mø (27, 54). After particle internalization and entrapment into a phagocytic vesicle, its content progressively acidifies. The phagosome will ultimately fuse with lysosomes, with subsequent acquisition and activation of cathepsins, culminating in the total digestion of the phagolysosomal content (31). In addition, cathepsins were associated with processing of microbial antigens as well as antigen presentation machinery to generate effective MHC–antigenic peptide complexes priming the adaptive immune response (11, 50). It is conceivable that pathogens evolved strategies to manipulate these early events in order to avoid the activation of the microbicidal mechanisms and survive within these cells that otherwise would destroy them. This is the case for Mtb and HIV. Both pathogens manipulate the microbicidal mechanisms of Mø to establish chronic intracellular niches. In the case of HIV infection, the cleavage and processing of viral proteins for the assembly of new virus particles are performed by the host cathepsin B (55). An inhibition of this process impairs the infectivity of nascent virions and cell-to-cell spreading, keeping virus infection undetected by the immune system (56–58). This may account for the chronic infection in Mø limiting the spread of new viruses in normal conditions. For Mtb, we found out early that during establishment of the infection, the pathogen downregulates most cathepsins and that, with the exception of cathepsin F, most of these proteases were implicated in pathogen killing (14).

Since cathepsin proteolytic activity is regulated by Csts (12), we investigated here the role of these protease inhibitors during infection of Mø with Mtb or HIV or during coinfection. Initially, we analyzed the gene expression of type I and II Csts during early events of infection. For *M. smegmatis*, a non-pathogenic species that is destroyed by Mø within 48 h, we found an early upregulation at 24 h of Csts A, C, F, and S. With the exception of CstSN, a Cst that is usually secreted out of the cell, all Csts showed a decreased gene expression relative to the first 24 h p.i. concomitant with the bulk destruction of bacteria in phagolysosomes (48). Thus, this may provide evidence that Csts, in physiological conditions, are operating to bring protease activity back to basal levels, following clearance of the bacteria. For Mtb at 24 h p.i., all Csts were downregulated except for CstA, which was similar to that observed for non-infected cells. Along the infection, Csts A, C, F, and SN had the most significant increases of gene expression, coincident with intracellular bacillus multiplication. Likewise, we noticed that the highest gene expression increases after 24 h p.i. for Csts A, C, and SN in mono-infection with Mtb or HIV, and coinfection with both pathogens. Since CstSN displayed an increased gene expression upon challenge with the non-pathogen *M. smegmatis*, it became less interesting to us for further investigation compared with Csts A and C, the most prominent hits in the context of pathogenic infection. Among the latter two candidates, while CstA accumulates more in the cytosol and in the nucleus (27, 28), CtsC tends to be trafficked to the endocytic pathway (29, 30). This indicates that CstC could be an important target for pathogens that co-localize within the same compartments.

To further examine the impact of increased CstC expression along infection, we performed siRNA silencing of CstC in Mø prior to infection. We found that siRNA silencing of CstC had a significant antimicrobial effect against Mtb either during mono-infection or during coinfection with HIV, leading to significant reduction of CFU similar to that obtained with PZA treatment, a first-line antibiotic against Mtb. An improved killing effect following CstC depletion was also observed during infection with clinical Mtb strains including a MDR-TB. As the silencing did not affect cell death, nor alter the internalization of bacilli, we infer that the impact on bacterial killing was attributed to a direct decreased inhibition of lysosomal enzymes. CstC is a potent inhibitor of most cathepsins including cathepsins B, L, and S; and it has been shown to accumulate in endolysosomal compartments (29, 30). Our results indicate that the silencing of this inhibitor strongly impacts cathepsin proteolytic activity, suggesting a direct effect in the very same compartment that usually contains Mtb and, therefore, contributing to increased pathogen killing. This is particularly relevant for cathepsin S, which is active across a broad pH range (16) and strongly contributes, through the phagolysosomal system, to kill intracellular bacilli (14, 15).

Previous studies have demonstrated CstC antiviral role *via* an inhibitory effect on viral proteases. In line with these evidences, CstC has been found to interfere with coronavirus replication in human lung cells (59), and in herpes simplex virus in human submandibular–sublingual and parotid cells (60), as well as with HIV in *in vitro* assays (61). An abnormal activity of CstC was found to target IdeS, the IgG cleaving protease of *Streptococcus pyogenes*; rather than acting as inhibitor, it enhanced IdeS activity (62). During infection with parasites, in a murine model of *leishmaniasis*, CstC was associated with T-cell conversion from Th1 into Th2, skewing the host immune system to favor parasite propagation by inducing the secretion of the immunosuppressive IL-10 (63). Our results in HIV-infected cells lead us to propose that the increased CstC expression observed during HIV infection will affect viral spread through an inhibitory effect on viral proteases, or by affecting cathepsin activity required to process virus particles. This will contribute to maintain virus infection silenced from immune surveillance, while maintaining provirus integrated in the host genome. In fact, for HIV, CstC silencing was translated into a significant impact on OmniCathepsin enzymatic activity (albeit not as prominent as for Mtb) with a higher magnitude than measured for cathepsin S alone during Mtb infection. Altogether, our results suggest these pathogens have evolved an interesting strategy to inhibit protease activity and enhance their intracellular survival and spread, or perhaps to remain undetected within infected cells against immunosurveillance.

CstC, as a cathepsin S inhibitor and regulator, plays a pivotal role in the control of cleavage and removal of the MHC class II invariant chain (Ii) (29, 50). It also downregulates the MHC-II chaperon H2-DM, resulting in diminished MHC-II–peptide presentation and reduced T-cell proliferation (64). CstC and cathepsin S have been shown to contribute to MHC class II antigen processing and presentation (11, 64). Here, we demonstrated that the silencing of CstC induces a significant increased expression of HLA class II at the cell surface during Mtb infection and coinfection with HIV, but not during HIV mono-infection. This is in accordance with the increased cathepsin S activity during bacterial infection. This translated into a better priming of CD4^+^ T lymphocytes in terms of high proliferation and increased IFN-γ secretion. All these results support our previous findings showing that by enhancing cathepsin S activity, a better priming of T cells by infected Mø can be achieved (15). The observed increase of IFN-γ secretion will certainly lead to proinflammatory activation of Mø with enhanced microbicidal activity against Mtb (31). IFN-γ may also contribute to control inflammation during active TB in accordance with previous studies showing that it inhibits the release of IL-1β and probably reduces lung immunopathology (65).

Finally, we also show that silencing CstC could significantly impact the adaptive response induced by infection with the BCG, indicating that modulation of its expression may improve vaccination approaches. Previously, it was shown that the IL-10-dependent inhibition of cathepsin S observed in BCG led to decreased vaccine capacity (66). Moreover, a recombinant BCG strain expressing active cathepsin S was able to overcome the inhibitory effects induced by IL-10 (67). Altogether, this suggests the potentiality of modulating cathepsin S activity by overexpression of the protein or by CstC depletion to strengthen the adaptive immune responses to infection.

Overall, the results indicate CstC as a potential therapeutic target in the Mø control of Mtb infection, which may also be proposed as a target in the context of Mtb/HIV coinfection. Here, we open new avenues for the development of future drug delivery systems for siRNA-based depletion of CstC in infected cells. Their inclusion in nanoparticles or liposomes targeting Mø receptors would allow their specific delivery to these immune cells and to concentrate them in the intracellular milieu. The resulting interference with CstC will improve cathepsin intracellular activity, overcoming the pathogen-induced blockade. Thus, CstC by restoring protease activity/inhibition balance emerges as an important new target to control infection. Also, microorganisms that depend on cellular proteases and their inhibitors might provide a solid frame for future research not only to better understand cathepsins/Cst function on pathogen replication and survival but also particularly to establish new therapeutic interventions where conventional antimicrobials have lost their efficacy.

## Data Availability Statement

The raw data supporting the conclusions of this article will be made available by the authors, without undue reservation.

## Author Contributions

Conceptualization: EA, DP, GL-V, CV, and ON. Methodology, acquisition, and analysis: DP, EA, CV, and JA-P. Investigation: DP, TV, MC, and MM. Resources: MJC. Editing: GL-V, CV, DP, and JA-P. Writing and editing: EA. Supervision and funding acquisition: EA. All the authors read, commented, and approved the final version of the manuscript.

## Funding

This study was supported by grants from the National Foundation for Science, FCT Fundação para a Ciência e Tecnologia – Portugal, PTDC/SAU-INF/28182/2017 to EA, UID/DTP/04138/2019 (to IMed-ULisboa).

## Conflict of Interest

The authors declare that the research was conducted in the absence of any commercial or financial relationships that could be construed as a potential conflict of interest.

## Publisher’s Note

All claims expressed in this article are solely those of the authors and do not necessarily represent those of their affiliated organizations, or those of the publisher, the editors and the reviewers. Any product that may be evaluated in this article, or claim that may be made by its manufacturer, is not guaranteed or endorsed by the publisher.
