# Supplementary figures and images for: Modulation of Cystatin C in Human Macrophages Improves Anti-Mycobacterial Immune Responses to Mycobacterium tuberculosis Infection and Coinfection With HIV
Source: Front Immunol. 2021 Nov 18;12:742822. doi: 10.3389/fimmu.2021.742822 (PMC8637326; doi:10.3389/fimmu.2021.742822)

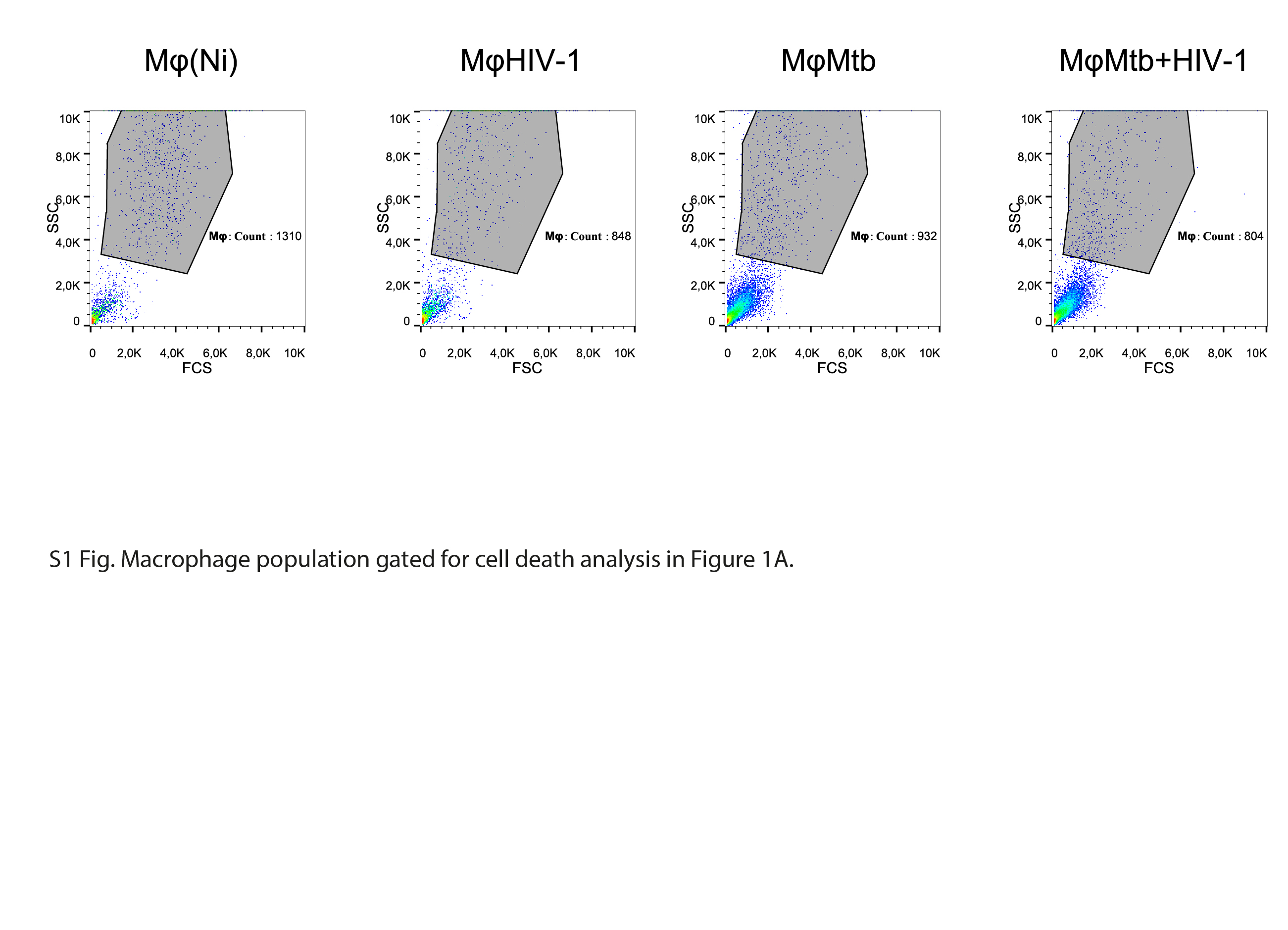

Supplement: Supplementary file 1 [file Image_1.jpeg]

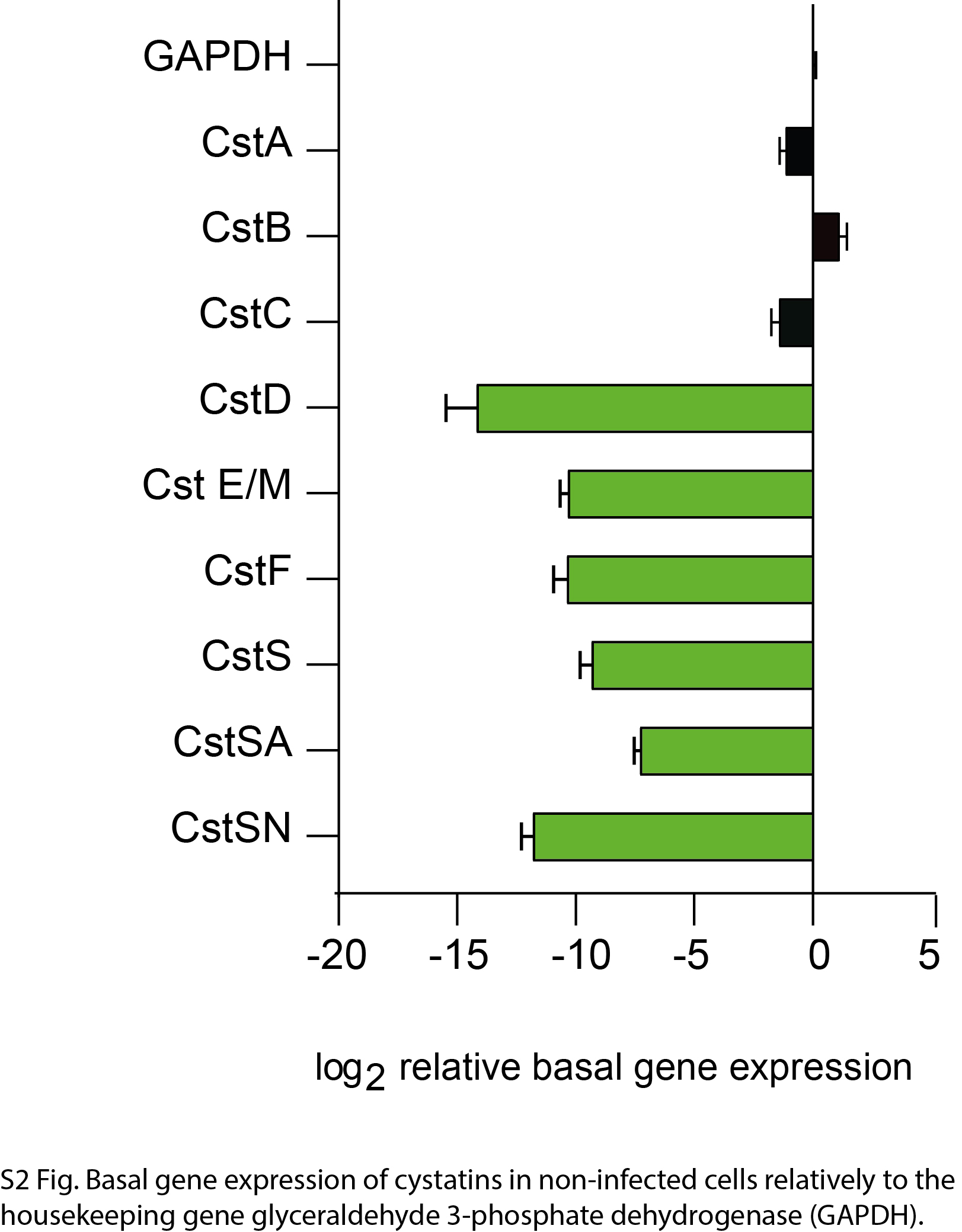

Supplement: Supplementary file 2 [file Image_2.jpeg]

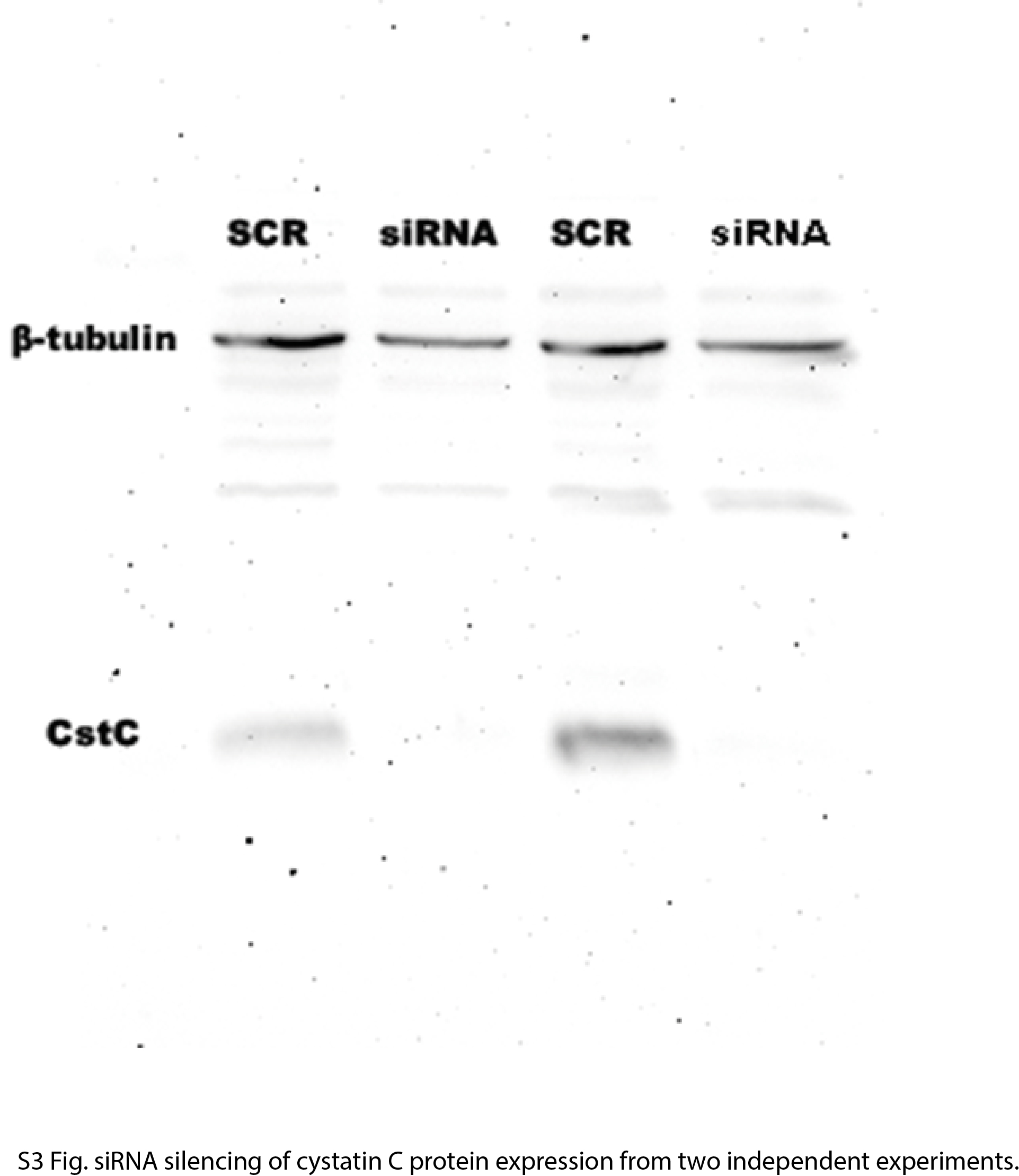

Supplement: Supplementary file 3 [file Image_3.jpeg]

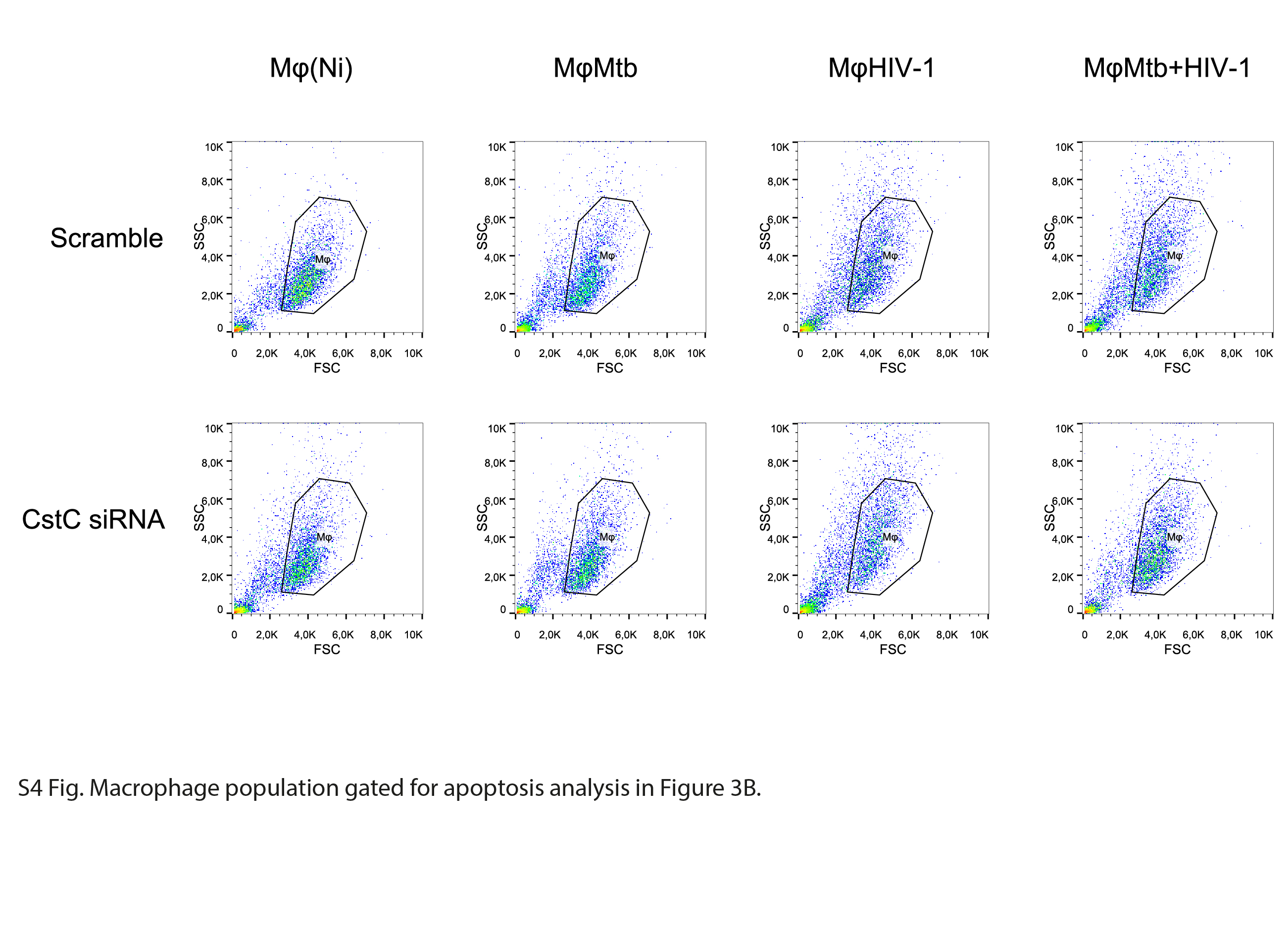

Supplement: Supplementary file 4 [file Image_4.jpeg]
